# Developing a Decision Aid to Facilitate Informed Decision Making About Invasive Mechanical Ventilation and Lung Transplantation Among Adults With Cystic Fibrosis: Usability Testing

**DOI:** 10.2196/21270

**Published:** 2021-04-14

**Authors:** Katherine L Dauber-Decker, Melissa Basile, D'Arcy King, Jennifer Polo, Karina Calise, Sundas Khan, Jeffrey Solomon, Daniel Dunne, Negin Hajizadeh

**Affiliations:** 1 Donald and Barbara Zucker School of Medicine at Hofstra/Northwell Feinstein Institutes for Medical Research Center for Health Innovations and Outcomes Research Manhasset, NY United States; 2 School of Health Professions and Human Services Hofstra University Hempstead, NY United States; 3 iDEAL Institute Loyola Marymount University Los Angeles, CA United States

**Keywords:** usability, medical informatics, clinical decision support, cystic fibrosis, advance care planning

## Abstract

**Background:**

Cystic fibrosis (CF) is a life-limiting genetic disease that causes chronic lung infections. We developed an internet-based decision aid (DA) to help patients with CF make better informed decisions regarding treatments and advance care planning. We built the DA around two major treatment decisions: whether to have a lung transplant and whether to agree to invasive mechanical ventilation (intubation).

**Objective:**

This study aims to conduct usability testing of the InformedChoices CF DA among key stakeholder groups.

**Methods:**

We performed a patient needs assessment using *think-aloud* usability testing with patients with CF, their surrogates, and CF clinicians. *Think-aloud* participants provided feedback while navigating the DA, and after viewing, they answered surveys. Transcripts from the *think-aloud* sessions and survey results were categorized into common, generalizable themes and optimizations for improving content, comprehension, and navigation. We assessed the ease of use of the DA (System Usability Scale) and also assessed the participants’ perceptions regarding the overall tone, with an emphasis on emotional reactions to the DA content, level of detail, and usefulness of the information for making decisions about either intubation or lung transplantation, including how well they understood the information and were able to apply it to their own decision-making process. We also assessed the DA’s ease of navigation, esthetics, and whether participants were able to complete a series of usability tasks (eg, locating specific information in the DA or using the interactive survival estimates calculator) to ensure that the website was easy to navigate during the clinic-based advance care planning discussions.

**Results:**

A total of 12 participants from 3 sites were enrolled from March 9 to August 30, 2018, for the usability testing: 5 CF clinicians (mean age 48.2, SD 12.0 years), 5 adults with CF, and 2 family and surrogate caregivers of people with CF (mean age of CF adults and family and surrogate caregivers 38.8, SD 10.8 years). Among the 12 participants, the average System Usability Scale score for the DA was 88.33 (*excellent*). *Think-aloud* analysis identified 3 themes: functionality, visibility and navigation, and content and usefulness. Areas for improvement included reducing repetition, enhancing comprehension, and changing the flow. Several changes to improve the content and usefulness of the DA were recommended, including adding information about alternatives to childbearing, such as adoption and surrogacy. On the basis of survey responses, we found that the navigation of the site was easy for clinicians, patients, and surrogates who participated in usability testing.

**Conclusions:**

Usability testing revealed areas of potential improvement. Testing also yielded positive feedback, suggesting the DA’s future success. Integrating changes before implementation should improve the DA’s comprehension, navigation, and usefulness and lead to greater adoption.

## Introduction

### Background

Cystic fibrosis (CF) is a life-limiting, progressive genetic disease that causes chronic lung infections [1,2] and persistent symptoms, including coughing, pneumonia, bronchitis, wheezing, difficulty breathing, and lack of weight gain and growth [3]. The average life expectancy for a person with CF is currently estimated at approximately 37 years [4]. However, because of variability among patients related to the natural course of pulmonary decline, it is difficult to estimate prognoses [5-10]. Therefore, it is often unclear when clinicians should initiate advance care planning (ACP) discussions with patients with CF. ACP allows patients’ early consideration of the kind of end-of-life care they may want while they are able to fully understand the implications of different treatment options. ACP is recommended by the American College of Chest Physicians [11]; however, it is not widely practiced in patients with CF [12]. Encouraging patients to plan their care is important so that their end-of-life desires and needs are fully acknowledged and protected.

**Figure 1:**
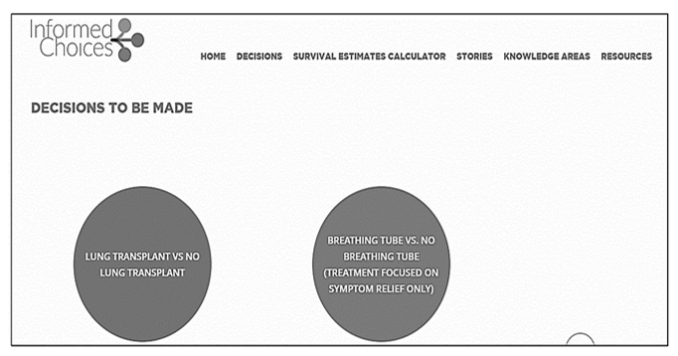
Cystic fibrosis internet-based decision aid—decisions page.

As part of the Cystic Fibrosis Foundation initiative to foster innovative approaches in CF-specific palliative care, our team at the Center for Health Innovations and Outcomes Research at Northwell Health undertook a multiphased study to develop an internet-based patient decision aid (DA) called InformedChoices [13]. We developed DA content around 2 crucial decisions that advanced patients with CF commonly face as their condition progresses: whether to have a lung transplant and whether to agree to intubation (invasive mechanical ventilation [IMV]) in the event of acute respiratory failure (Figure 1). The goal of the DA is to increase preference-congruent care at the end of life for patients with advanced stage CF by fostering shared decision-making conversations among adults with CF, their clinicians, and family caregivers. Therefore, the purpose of the DA is to be used by the CF clinician with their patients with CF and family members during outpatient clinic visits. The development of our DA content was guided by several key bodies of literature—DA design—specifically the International Patient Decision Aid Standards (IPDAS) Collaboration criteria for DA design, which presents a checklist of quality standards for the development of DA content [14]. For example, the IPDAS criteria provides patients a range of visual options for viewing prognostic survival estimates. Therefore, we included icon arrays, percentages, and graphs to convey information on the prognostic outcomes [15]. IPDAS also encourages the inclusion of methods to clarify patients’ values and goals of care. This is known as *preference elicitation.* Previous work encourages interactive and hierarchical approaches to eliciting preferences [16-18]. Therefore, we chose an interactive exercise offering patients a range of possible outcomes related to both lung transplantation and IMV. For each risk and benefit offered, users are able to slide a tab along a continuum from not important to very important. Finally, patients could view their results with the risks and benefits placed in hierarchal order from most important to least important. Additional criteria that we considered when designing the DA and on which we focused our usability testing included the use of plain language that could be understood by end users of various educational backgrounds, using stories or narratives that represent a range of outcomes, and presenting information in a balanced manner. Regarding the last point, when offering the risks and benefits of the various treatment options, we presented this information in side-by-side columns to allow clear visual representation of the risks and benefits.

We also explored the literature on both current DA development specific to ACP decision making [19-21] and literature on specific ACP and palliative care concerns faced by people with CF [22,23]. From this literature, we learned that individuals incorporate various types of knowledge into their decision making and often draw on previous lived experiences, which may *compete* with the biomedical information being conveyed. This influenced the study design of our usability testing, that is, the extent to which competing knowledge frameworks may actually impact users’ ability to understand the biomedical information being conveyed. This is reflected in our usability testing questions, which seek to determine the extent to which users not only *understood* the information but were then able to apply it to their own medical condition. Finally, there is a more recent body of literature on developing models of primary palliative care for CF. The focus is on allowing CF care teams to offer basic palliative care services, including ACP and goals of care discussions to people with CF on an ongoing basis, throughout the life course [22,23].

### Objectives

Our DA is meant to be used in such contexts, that is, shared decision making among patients with CF, CF providers, and family and surrogate caregivers. Therefore, one of our usability testing goals was to ensure that CF clinicians were comfortable conveying the information contained in the DA about advanced CF treatments and that patients and caregivers could understand the information. We also sought to assess the possible emotional reactions to the information among patients and their caregivers. Usability testing allowed us to assess these factors with our target end users before rollout of a larger scale feasibility and acceptability study undertaken in outpatient clinic settings.

Finally, the design of the DA was further informed by a qualitative needs assessment where we interviewed adult patients with CF and family caregivers about their information needs as they pertained to ACP and CF treatment decisions and any previous discussions with their clinicians about both intubation and lung transplantation [13]. We also informally surveyed CF clinicians, asking them to tell us what information they felt their patients needed to know to make an informed decision about both intubation and transplant and to provide us with relevant peer-reviewed articles on which to base DA content. Guided by the abovementioned IPDAS criteria, review of literature, and direct stakeholder engagement, the DA’s website content includes descriptions of both intubation or IMV and lung transplantation, including the risks and benefits of each procedure. We also provided tailored prognostic estimates using multiple displays of data to accommodate different levels of health numeracy and preferences for information styles [24]. The needs assessment revealed that several participants preferred to learn about IMV and lung transplantation by hearing directly from patients with CF who had experienced intubation or lung transplantation. They expressed a desire for a more personal connection, that is, to know *what it felt like* to go through lung transplant or IMV, as opposed to the more clinical descriptions of the procedures that they were often given by their providers. This type of information allowed for greater emotional engagement with the DA content, which we believe may appeal to certain individuals’ learning styles and preferences for information. Therefore, we conducted interviews with patients with CF or family members about these treatments and edited them for inclusion in the DA. We also included additional content areas covering CF-specific mental health care, palliative care, and ACP based on what CF clinicians believed to be important for informed decision making related to CF ACP. Furthermore, from the needs assessment, we discovered that people’s desire for information varied, with some people wanting to know very detailed information about their treatment options and others preferring to know less. On the basis of this, our DA design allowed for basic as well as detailed information, as we allow individuals to navigate to a resources page that contains all of the references we used to write DA content (to accommodate those with high information-seeking preferences) and preference elicitation exercises for both IMV and lung transplantation, per IPDAS guidelines. Our overall goal was to ensure that our DA could accommodate a wide array of learning styles and information preferences to ensure the uptake of the information presented.

Following the initial design of the DA, we performed usability testing to maximize adoption, comprehension, and end user benefit before the final phase of the study—feasibility and acceptability testing of the DA among adults with CF, providers, and family members in ACP shared decision-making conversations in outpatient clinic settings. Although the DA is intended for shared decision-making conversations, our focus in undertaking a usability testing phase was to assess, among the 3 key stakeholder groups (patients, clinicians, and family surrogate caregivers), individual-level comprehension of the written content; perceptions of the usefulness for communicating about the risks and benefits of both pursuing or not pursuing lung transplant; and accepting or refusing intubation, visibility, and ease of navigating the website. Our intention was to ensure that we had addressed any potential design problems and that content was understandable before the rollout of a larger scale feasibility and acceptability study. Herein, we present the results of the testing conducted among key CF stakeholders.

## Methods

### Study Design and Data Collection

Eligible participants were clinicians, patients, and surrogate caregivers who met the criteria described in the *Eligibility* section. On enrollment, each participant completed a basic demographic and health survey. Participants were then shown the DA either in person or remotely via Webex, a Health Insurance Portability and Accountability Act–compliant web-based conferencing platform. In both scenarios, a member of the research team observed the process and took detailed notes. Participants were asked to navigate through the DA at their own pace and click on the pages in any order they wished. Participants were encouraged and reminded throughout the testing session to *think aloud* as they progressed through the content and to voice their comments and reactions to the information and images in real time. This process was captured using Hypercam (Microsoft), a screen capture and audio recording software. Once participants viewed the DA, they were asked to complete 3 questionnaires to elicit their postexperience feedback. First, the validated System Usability Scale (SUS) [25] was used to measure the ease of use of the DA. The next 2 questionnaires were developed specifically to assess this specific CF DA. One questionnaire asked open- and closed-ended questions designed to measure the participants’ perceptions of the overall tone, with an emphasis on emotional reactions to DA content (eg, personalized prognostic estimates indicating survival over a 3-year period and reactions to images of an intubated patient), level of detail, and usefulness of the information for making decisions about either intubation or lung transplantation, including how well people understood the information and were able to apply it to their own decision-making process. This questionnaire also addressed the ease of navigation and esthetics of the DA. The other questionnaire focused on having participants complete a series of usability tasks (eg, locating specific information in the DA or using the interactive survival estimates calculator) to ensure that the website was easy to navigate during the clinic-based ACP discussions (Multimedia Appendices 1-4). We also administered participant demographics surveys (Multimedia Appendix 5 and Multimedia Appendix 6). All questionnaires were administered directly via REDCap, where the responses were stored, anonymized, and exported to Excel for analysis. All Hypercam recordings were transcribed for qualitative analysis. Feedback from the surveys and recordings were coded into usability themes, as described in *Data Analysis* section.

### Eligibility

#### Clinician Participants

Doctors, other advanced practice providers (nurses, nurse practitioners, and respiratory therapists), or social workers who treat patients with CF aged >18 years were eligible for the study.

#### Patients

Patients with lung function score of forced expiratory volume in the first second <55% and/or clinician’s assessment of moderate to advanced stage CF, who had already undergone lung transplant, who were aged >18 years, and who speak English were eligible.

#### Surrogate Caregiver Participants

English-speaking individuals aged >18 years and currently caring for patients who meet the inclusion criteria mentioned earlier and caregivers of patients who died within the year before enrollment were eligible. Caregivers were primary caregivers and decision makers for people with CF and either parents or significant others of adults with CF; however, they did not need to be related to the patients who were enrolled in the study (ie, we did not enroll patients and caregivers as dyads).

In addition, all those participating remotely were required to have access to a computer with internet capability and a web camera installed or attached to their computer.

### Recruitment and Consent

All participants were recruited from the Northwell Health CF Care Center, the University of Pennsylvania Perelman Center for Advanced Medicine, or the University of California San Diego Health Adult Cystic Fibrosis Program. Clinicians were recruited by the nonclinician members of the research team (ie, research coordinators) to avoid potential pressure to participate from their clinician peers who were members of the research team. Clinician-specific informed consent forms specifically stated that participation was voluntary and that decisions to not enroll in the study would not impact their employment. After being approached by a member of the research team at their respective sites, interested patients, surrogate caregivers, or clinicians were then referred to the research team at Northwell Health, the lead study site, where an investigator reviewed the main points of the study, answered any additional questions, and scheduled a time for the testing session. For in-person testing, written informed consent was obtained on site before initiating the testing session. For remote consent, the Northwell Health institutional review board–approved methods for remote consent were used. This involved using a phone script and sending consent forms via email before the scheduled testing day.

All participants received US $100 compensation for their time, regardless of their stakeholder groups. Before the initiation of our study, we obtained approval from the Northwell Health institutional review board. The funding agency had no role in the design of the study.

### Data Analysis

Audios from the Hypercam recordings were transcribed and analyzed qualitatively by the Northwell Health Usability Lab to identify usability issues, including whether users were able to complete assigned tasks, and to identify any barriers encountered (eg, whether content was understood and whether users were able to navigate efficiently through the DA). Usability Lab members performed a thematic analysis of the transcripts from the Hypercam recordings. This involved coding the transcripts into the following themes: functionality, visibility and navigation, and content and usefulness. Members of the Usability Lab first met to ensure that all readers were coding in a similar fashion and establish interrater reliability. This was established through discussion following the individual coding of a subset of transcripts. Each transcript was then coded and analyzed by a member of the Usability Lab, and a summary of common suggestions for each theme was generated. Usability Lab members brainstormed and discussed changes that could be made to the website to address common issues and suggestions, which were then incorporated into a subsequent round of DA revisions.

Data from closed-ended questions administered during testing were summarized descriptively. Our sample size was limited to 12 participants; therefore, we were unable to perform rigorous statistical analyses. As the established rule of usability testing states that 5 participants are sufficient to detect 80% of a product’s usability issues [26], we chose a total sample of 12 participants, including 5 patients with CF, 5 clinicians, and 2 surrogate caregivers.

## Results

### Participant Characteristics

A total of 12 participants from 3 sites were enrolled from March 9 to August 30, 2018. Our sample included 5 CF clinicians (physicians, social workers, and nurse practitioners), 5 adults with CF (2 of whom had already undergone a lung transplant), and 2 family and surrogate caregivers of people with CF. A summary of the participants’ demographic characteristics is available in Table 1.

**Table 1:** Participants’ demographic characteristics.

Participant				Value
**Clinicians (n=5)**				
	Age (years), mean (SD)		48.2 (12.0)	
	Gender (female), n (%)		5 (100)	
	Years of experience with patients with cystic fibrosis, mean (range)		17.6 (9-29)	
	**Profession, n (%)**			
		Physician	2 (40)	
		Nurse practitioner	1 (20)	
		Social worker	2 (40)	
**Patients and surrogates^a^ (n=7)**				
	Age (years), mean (SD)		38.8 (10.8)	
	Gender (female), n (%)		3 (40)	
	**Role, n (%)**			
		Patient	5 (70)	
		Surrogate	2 (30)	

^a^For the patients and surrogates group, 5 of the 7 participants provided age and gender information.

### Quantitative Analysis of Questionnaire Responses

Our quantitative analysis was performed using the SUS [25] to assess the usability of the DA. Among the 12 participants, there was an average SUS score of 88.33, which indicates an *excellent* score of *B* on the scale. The 5 clinicians gave the tool an average SUS score of 89.5; the 7 patients and surrogates gave an average score of 87.5. A summary of each participant’s SUS scores is presented in Table 2.

**Table 2:** System Usability Scale scores.

Participant number	Participant category	System Usability Scale
1	Clinician	82.5
2	Clinician	90
3	Clinician	100
4	Clinician	92.5
5	Clinician	82.5
6	Surrogate	90
7	Patient	100
8	Patient	100
9	Patient	92.5
10	Surrogate	90
11	Patient	47.5
12	Patient	92.5
—^a^	—	88.33

^a^The average System Usability Scale score for all participants is presented in the last row. This does not imply missing data.

### Thematic Analysis of Questionnaires and Think-Aloud Hypercam Recording Responses

Participants’ comments from all *think-aloud* testing sessions and surveys were grouped into 3 overarching themes: functionality, visibility and navigation, and content and usefulness. Major suggestions from these themes and accompanying participant quotes from the surveys and session transcripts are summarized in Table 3 and in the following sections.

**Table 3:** Summary of participants’ observations and comments and solutions to be implemented, grouped by usability theme.

Usability theme and participants’ observations and comments^a^			Solutions implemented
**Functionality**			
	The Breathing Tube and Lung Transplant page drawers do not flow logically: “Situations in which a CFb patient... may need to decide about a breathing tube for procedures...this might go first...before we even look at the risks and benefits.” (Patient)	Rearrange drawers (Figure 2): Describe why it is important to think about getting a breathing tube or lung transplant Discuss factors associated with good and poor prognoses Provide more information about the treatment option and situations in which a patient may need to decide about the treatment List risks and benefits associated with the treatment option	
**Visibility and navigation**			
	The details in the pictures showing intubation and tracheostomy are difficult to see: “I wish I could see a bit more detail.” (Patient) “Add [an] enlarge feature to read the small labels.” (Patient)	Add an enlarge feature to the images	
	The risks and benefits sections of the Breathing Tube and Lung Transplant pages are repetitive: “I would take away the repetitive risks vs benefits tables for each procedure.” (Clinician) “...possibly revamping the pro and con section so that it doesn’t have so much repeating info throughout.” (Patient)	Condense the risks and benefits sections of these pages and eliminate the repetition	
	Participants were unclear on whether the survival estimates calculator provides estimates for before or after lung transplant or intubation: “I actually took the estimates to mean posttransplant, so I feel like I would need to carefully clarify with the patients.” (Clinician)	Visually emphasize the statement at the top of the page telling users that estimates are for before treatment by bolding the text and enlarging the font size (Figure 3)	
	Participants were concerned that some patients might take prognosis estimate percentages too literally: “I just think that the concrete thinkers...could have a difficult time with that information even though you explained that they’re estimates and how you got the estimates...that it's not written in stone. I think those concrete thinkers you know would...possibly have a little difficulty with that.” (Clinician)	Emphasize the following statement by bolding the text: “Remember these are only estimates and the numbers may not apply specifically to you” (Figure 4)	
	Users need to scroll all the way back to the top of the Stories page to exit a story: “I chose to read the transcripts and when I got to the end of the lengthy transcript I had to scroll all the way to the top to X out of the story.” (Clinician)	Add a cancel button and close window option to the bottom of each story	
**Content and usefulness**			
	Patients with CF are motivated by their desire to survive for their children: “Another thing that could be a question to ask in this area and it isn't pertinent to everybody but it sure has been pertinent to a lot of our patients who are considering transplant...they want to be around as long as they can be for their children.” (Clinician)	Add the following phrase to the What’s Important to Me slider: “Seeing my children grow up is important to me” (Figure 5)	
	Posttransplant pregnancy can pose challenges to both a mother with CF and their fetus: Clinicians are trying to improve the process of explaining to patients that they “...can’t physically carry [children themselves] but we can have [them] meet with an OBGYN or fertility providers before transplant to give [them] the best possible outcomes of having children in some other way or even...counseling about adoption..., surrogacy, different things like that.” (Clinician)	Add descriptions of alternative options for becoming a parent, including adoption and surrogacy, to the decision aid (Figure 6)	

^a^The quotations in this table were obtained from participant surveys and *think-aloud* transcripts. Participant categories are indicated in parenthesis following each quotation (ie, clinician, patient, or surrogate).

^b^CF: cystic fibrosis.

**Figure 2:**
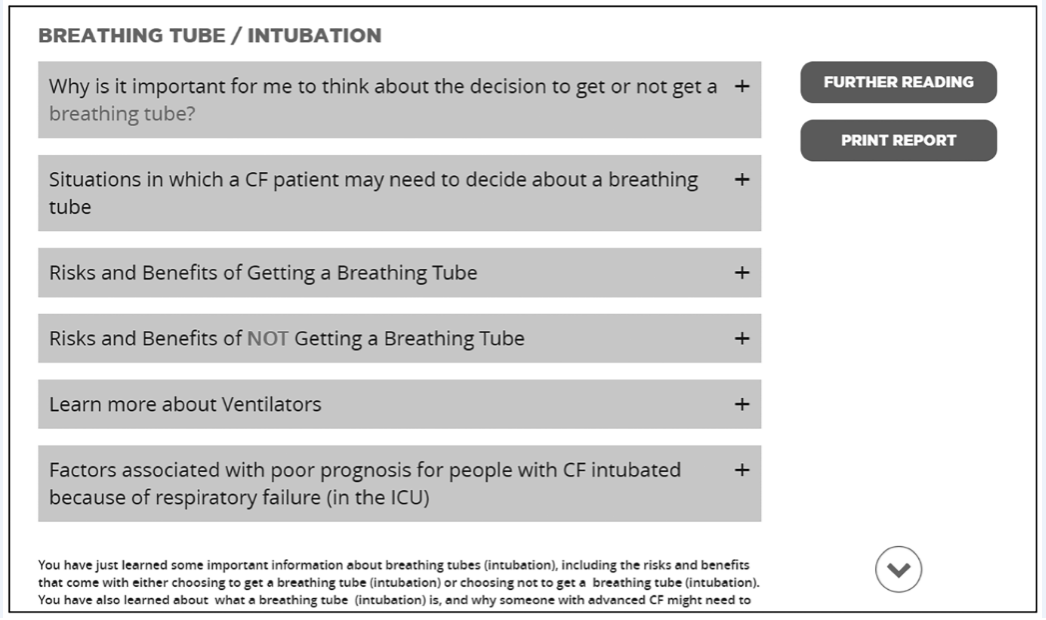
Areas recommended for improvement on the cystic fibrosis decision aid. Drawer design as seen by study participants. Drawers on the Breathing Tube/Intubation (shown) and Lung Transplant (not shown) pages have now been reordered as follows: (1) why it is important to think about getting a breathing tube or lung transplant, (2) factors associated with good and poor prognoses, (3) more information about the treatment option, (4) situations in which a patient may need to decide about the treatment, and (5) risks and benefits associated with each treatment option. CF: cystic fibrosis; ICU: intensive care unit.

**Figure 3:**
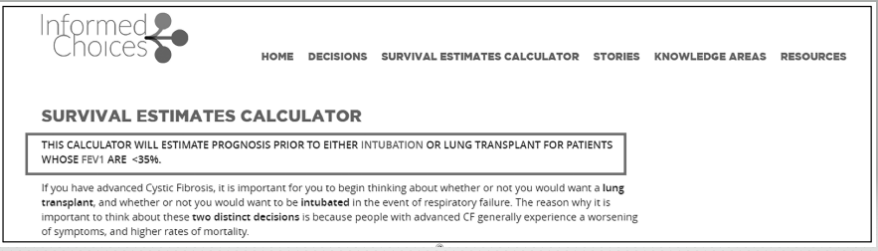
Survival estimates calculator. The initial phrase explaining the survival estimates calculator has been visually emphasized by bolding and enlarging the font. CF: cystic fibrosis.

**Figure 4:**
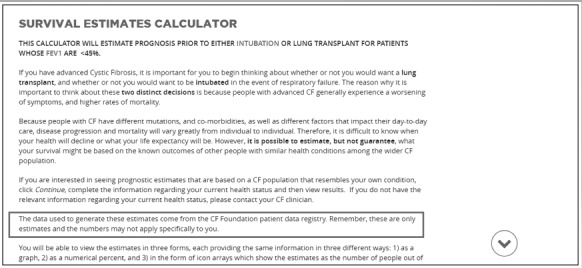
Survival estimates calculator page as seen by study participants. The statement that the percentages generated by the survival estimates calculator are only estimates and do not necessarily apply to individual patients has now been bolded for emphasis. CF: cystic fibrosis.

**Figure 5:**
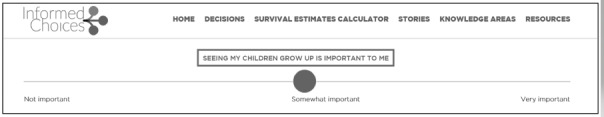
The addition of the phrase “Seeing my children grow up is important to me” to the What’s Important to Me slider will increase the usefulness of the slider for those who want to survive for their children.

**Figure 6:**
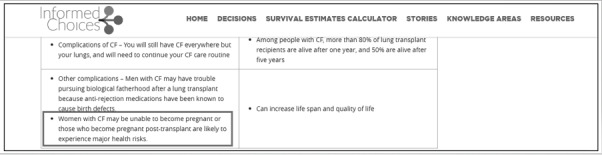
Pregnancy information as seen by study participants. Information about adoption and surrogacy has now been added to explain alternatives to pregnancy. The boxes indicate areas where text is being changed or emphasized to address user feedback. CF: cystic fibrosis.

#### Functionality

First, the changes in functionality were identified. For example, one suggestion involved the drawer design of the Breathing Tube and Lung Transplant pages. A drawer design helps to minimize the content to prevent the user from seeing too much text at one time and becoming overwhelmed. By expanding each *drawer* category, the user has the ability to view additional content of interest. One suggested optimization was that the drawers on the Breathing Tube and Lung Transplant pages should be reordered to improve the logic of the DA’s flow. We have reordered the drawers, accordingly, as shown in Table 3 (Figure 2). Reordering the topics to make the flow of information more logical should make content more accessible and improve individual-level comprehension.

#### Visibility and Navigation

We also identified areas for improvement in the visibility of CF DA. For example, participants suggested that we enlarge the pictures displaying intubation and tracheostomy to increase the visibility of the smaller details of the images. One participant suggested that we:

add [an] enlarge feature to read the small labels.

With this change, users’ ability to engage with this content should improve.

In addition, several participants pointed out the repetition in the risks and benefits sections of the Breathing Tube and Lung Transplant pages. One participant from the patient and surrogate group suggested:

...possibly re-vamping the pro and con section so that it doesn’t have so much repeating info throughout.

Condensing this section should eliminate repetition.

Next, several users were unclear on whether the survival estimates calculator provided patients with estimates before or after lung transplantation or intubation. In a survey response, one clinician said:

I actually took the estimates to mean post-transplant...

Accordingly, we have bolded the text and enlarged the font size of the statement at the top of the page, telling users that these are pretreatment estimates (Figure 3). In addition, there was concern among clinicians that some patients might take these percentages too literally. One of our clinician participants said:

*I just think that the concrete thinkers...could have a difficult time with that information even though you explained that they’re estimates and how you got the estimates...that it's not written in stone.*

On the basis of this feedback, we have emphasized the following statement by bolding the text: “Remember these are only estimates and the numbers may not apply specifically to you” (Figure 4). These changes should help with users’ emotional responses to and individual-level understanding of the prognostic estimates so that they can better understand and use this information.

Finally, our testing revealed an area for improvement in navigation. One clinician participating in our testing referred to a navigation issue on the patient and caregiver Stories page. The clinician said:

I chose to read the transcripts and when I got to the end of the lengthy transcript, I had to scroll all the way to the top to X out of the story.

As a result, the navigation on this page has been amended with the addition of a cancel button or close window option to the bottom of each story, rather than the requirement that users scroll back to the top of the page to close each of the individual stories.

Although we identified areas in which to improve visibility and navigation, our usability testing participants’ ability to navigate to the tasks was already excellent. When asked whether they were able to navigate to the pages containing information about lung transplants, the patient and caregiver stories, and the What’s Important to Me slider, all clinicians were able to do so. In addition to page navigation, all clinicians were able to complete the What’s Important to Me slider and view their results. Finally, 4 of 5 clinicians (80%) were able to find the resources for making an advance directive (Table 4). The patients and surrogates were asked to complete the same tasks. All patients and surrogates participating in our testing were able to find the pages with information about lung transplants, the patient and caregiver stories, and the What’s Important to Me slider. All patients and surrogates were also able to complete the What’s Important to Me slider. Finally, 6 of the 7 patients and surrogates (86%) were able to find the resources for making an advance directive. Overall, the navigation of the site was easy for the clinicians, patients, and surrogates who participated in usability testing.

**Table 4:** Task completion exercises.

Question			Clinicians (n=5), n (%)		Patients and surrogates (n=7), n (%)
**Navigate to the page containing basic information about lung transplant. Were you able to complete this task?**					
	Yes	5 (100)		7 (100)	
	No	0 (0)		0 (0)	
**Find resources for making an advance directive. Were you able to complete this task?**					
	Yes	4 (80)		6 (86)	
	No	1 (2)		1 (14)	
**Find the page title “What’s Important to Me” for breathing tube. Were you able to complete this task?**					
	Yes	5 (100)		7 (100)	
	No	0 (0)		0 (0)	
**Complete the exercise and see your results. Were you able to complete the task?**					
	Yes	5 (100)		7 (100)	
	No	0 (0)		0 (0)	
**Find the page containing patient and caregiver stories about intubation and lung transplant. Listen to “Jeff’s Story.” Were you able to complete this task?**					
	Yes	5 (100)		6 (86)	
	No	0 (0)		1 (0)	

#### Content and Usefulness

Finally, several changes to improve the content and usefulness of CF DA were recommended. One clinician mentioned that patients with CF are often motivated by their desire to survive for their children. Accordingly, we have added the following phrase to the slider: “Seeing my children grow up is important to me” (Figure 5). In addition, as posttransplant pregnancy can pose challenges to both mother and fetus [27], one participant suggested that we include information about alternatives to childbearing, such as adoption and surrogacy, on the Lung Transplant page. The clinician said that in their work settings, they are trying to improve the process of explaining to patients that they:

...can’t physically carry [children themselves] but we can have [them] meet with an OBGYN or fertility providers prior to transplant to give [them] the best possible outcomes of having children in some other way or even...counseling about adoption..., surrogacy, different things like that.

These alternative options for becoming a parent have been added to the DA and should address an important emotional aspect of patient decision making (Figure 6).

## Discussion

### Principal Findings

Although both the clinician and patient and surrogate groups were largely able to complete each of the given tasks, our usability testing sessions revealed several areas for improvement on the CF DA, which we have incorporated. In the functionality theme, suggestions included reordering the content for a more logical flow. In the visibility and navigation theme, optimizations included enlarging the pictures, condensing sections to reduce repetition and improve clarity, visually emphasizing certain features, and adding additional cancel button or close window options to reduce unnecessary scrolling. Suggested improvements to content and usefulness included adding information about adoption and surrogacy for those who wish to become parents following lung transplantation.

In addition to their suggestions for ways to improve the CF DA, participants gave us positive feedback and felt that the DA would be of great benefit to future users. Notably, one of our participants, a surrogate who had children with CF and was also a nurse, said the following:

...I think it's a great tool. I think it’s good to have this discussion. Even on my job learning, we talk about lung transplant but it’s nice to have something to, you know, to open up the conversation.

One participant pointed to the DA’s completeness, describing it as:

Very well done, very clear, hits all important considerations people need to make.

Another participant from the patient and surrogate group stated:

...this website is very informative and it’s my belief that it will help a lot of people in the decision-making process.

Therefore, although there was room for improvement, participant responses point toward the future success of the DA in helping patients with CF and clinicians to make informed treatment decisions.

In designing the DA website content, one goal was to facilitate informed decision making via patient or clinician shared decision making. Previous work on informed decision making explores how to best present biomedically based information to ensure that those with low health literacy and numeracy can understand the information being presented. This correctly assumes that an informed decision rests on the individual-level understanding of the information being presented. Various studies have explored language levels (eg, *readability* should be at the eighth-grade level), and numeric data should be presented to ensure comprehension. Our previous work in DA design has further identified the importance of *uptake*, that is, the extent to which individuals are able to comprehend information and then apply it to their own decision making. In this way, our work adds to the literature on informed decision making by emphasizing patient-level self-assessment of what makes the patient similar to or different from the data being presented and thereby the extent to which the information is relevant to them. We were concerned with factors that may impact uptake, including previous lived experiences and emotional responses to the information. Therefore, our usability testing questions focused on assessing reactions to the tone of information about end-of-life and advanced CF treatment options. For example, all participants were asked the following survey question: “Was the tone of the information in the decision aid website appropriate?” Importantly, every participant answered “Yes” to this question. Similarly, participants were asked to comment on their reactions to seeing images of an intubated patient. None of the participants indicated emotional distress in their answers, and some even wanted to see the images in more detail. Taken together, participants’ overall feedback on the website combined with their responses to these questions eliciting emotional reactions to the website’s content indicated that the tone of the website was appropriate and would not elicit emotional responses that would interfere with their ability to comprehend and use the website’s content.

Our usability testing needs to be understood within the wider context of our multiphased study to develop and test the InformedChoices CF ACP DA. Beginning with a needs assessment, we sought to design a communication tool that asks people with a lifelong chronic illness to consider their future treatment choices in the event that their illness has progressed to the point at which they need to decide between life-extending treatment and comfort care. As a result, it was essential to assess both the functionality of the DA and individual-level reactions to the content in a controlled setting. Usability testing also allowed us to determine how comfortable clinicians would be accessing and communicating DA content to their patients with CF and how patients would react emotionally to the information before we undertook feasibility and acceptability testing within the context of an outpatient clinic setting, on a wide scale, across multiple sites.

### Next Steps

On the basis of the feedback from the usability testing, we revised the DA. We are currently undertaking multisite feasibility testing of the DA, where we are observing clinicians using it with patients with CF and surrogate caregivers during outpatient clinic visits. Following this, we will make additional revisions before rolling out the DA for use in our clinics and beyond. Our plan is to update the DA regularly as new information and treatments become available, including the survival estimates calculator as survival estimates change, and to add additional patient narratives.

### Limitations

One major limitation of our study is that we did not administer the SUS again after revisions were made to the DA. Ideally, we would hope to see an increase in the SUS score after making our changes to the website; however, this was not a part of our study. Another limitation is that we did not test the end user comprehension of the DA. Further analysis of end user response to the DA will be performed as part of a feasibility study in the future. This will consist of observing clinicians, patients, and family caregivers using the DA during 2 ACP conversations in outpatient settings, where we will measure feasibility and acceptability as well as changes in knowledge and decisional conflict over multiple time points. The sample size will also be larger for this phase of our study. Our usability study results are also limited by sample size; however, usability testing is often performed iteratively and with small samples to allow for more in-depth understanding of barriers to use. We are confident that our usability testing sample of 12 participants was large enough for us to obtain substantial feedback, as small sample sizes have been shown to be sufficient to detect most of the usability issues of a product [26]. However, the small sample size precluded us from performing statistical analyses of our survey response data. Another limitation comes from our highly health literate test population, including clinicians who treat patients with CF and well-informed patients with CF and their surrogates. It is possible that not all of our future end users will be as health literate as our usability testing participants; however, as the DA’s end users will be clinicians, patients with CF, and surrogates of patients with CF engaged in shared decision-making conversations, it is highly likely that the opinions of our test population provide an accurate representation of the views of our target audience.

### Conclusions

Usability testing helped us identify several areas for improvement of the CF DA. On the basis of user feedback, we have included these changes before implementation of the tool to improve the comprehension, navigation, retention, and overall usefulness of the DA. By integrating participant feedback and making these changes to the CF DA, we hope to improve the site in terms of end user benefits. We expect that these enhancements will lead to higher overall adoption rates of DA by clinicians, patients, and surrogates within our health system. We hope that in the future, this web-based clinical DA tool can be expanded for use in other health systems to help patients with CF and clinicians with ACP and the difficult decisions associated with CF.

### Practice Implications

We modified the CF DA based on the user feedback obtained from our usability testing. Integrating changes before implementation should improve the DA’s comprehension, navigation, and usefulness. Importantly, this should also lead to a greater adoption of the DA.

## Appendix

Multimedia Appendix 1 Surveys and measures used as part of the usability testing.

Multimedia Appendix 2 The system usability scale. The system usability scale is a 10-item questionnaire with 5 response options: strongly agree (5), agree (4), neither agree nor disagree (3), disagree (2), or strongly disagree (1).

Multimedia Appendix 3 Usability task completion exercises.

Multimedia Appendix 4 Usability testing survey.

Multimedia Appendix 5 Clinicians demographics.

Multimedia Appendix 6 Patient and surrogate demographics.

## Abbreviations

ACP: advance care planning
CF: cystic fibrosis
DA: decision aid
IMV: invasive mechanical ventilation
IPDAS: International Patient Decision Aid Standards
SUS: System Usability Scale
