# Supplementary material for: Developing a Decision Aid to Facilitate Informed Decision Making About Invasive Mechanical Ventilation and Lung Transplantation Among Adults With Cystic Fibrosis: Usability Testing
Source: JMIR Hum Factors. 2021 Apr 14;8(2):e21270. doi: 10.2196/21270 (PMC8082389; doi:10.2196/21270)
Supplement: Multimedia Appendix 1 [file humanfactors_v8i2e21270_app1.docx]

Appendix

Appendix 1. Surveys and measures used as part of the Usability Testing

| Questionnaire | Total Questions | Type of Questions |
| --- | --- | --- |
| Patient/Surrogate Demographics | 28 | Open and closed ended question covering basic socio-economic background and detailed information current CF health |
| Clinician Demographics | 10 | Open and closed ended questions covering basic demographics and details about current CF-specific clinical practice. |
| Usability Task Completion Exercise Questionnaire | 6 | Participants are instructed to complete 5 tasks related to navigating throughout the website, and then asked if they were able to complete each task. Responses: Yes or No questions |
| Usability Testing Survey | 39 | 28 multiple choice and Yes or No questions – 13 open/closed questions to elicit feedback from participants on DA content and design with sections covering: flow/ease of navigation, content/readability, and style and design |
| The System Usability Scale | 10 | Multiple choice (Strongly agree- Strongly disagree) |
