# Supplementary material for: Developing a Decision Aid to Facilitate Informed Decision Making About Invasive Mechanical Ventilation and Lung Transplantation Among Adults With Cystic Fibrosis: Usability Testing
Source: JMIR Hum Factors. 2021 Apr 14;8(2):e21270. doi: 10.2196/21270 (PMC8082389; doi:10.2196/21270)
Supplement: Multimedia Appendix 2 [file humanfactors_v8i2e21270_app2.docx]

Appendix 2. The System Usability Scale (SUS). The SUS is a 10 item questionnaire with 5 response options: Strongly Agree (5), Agree (4), Neither Agree or Disagree (3), Disagree (2) to Strongly Disagree (1).

1. I think that I would like to use this website frequently.
2. I found the website design unnecessarily complex.
3. I think the website will be easy to use.
4. I think that I would need the support of a technical person to be able to use this website.
5. I found the various functions in this website were well integrated.
6. I thought there was too much inconsistency in this website.
7. I would imagine that most people would learn to use this website very quickly.
8. I found the website’s workflow very cumbersome to use.
9. I would feel very confident using the website.
10. I needed to learn a lot of things before I could get going with this website.
