# Supplementary material for: Developing a Decision Aid to Facilitate Informed Decision Making About Invasive Mechanical Ventilation and Lung Transplantation Among Adults With Cystic Fibrosis: Usability Testing
Source: JMIR Hum Factors. 2021 Apr 14;8(2):e21270. doi: 10.2196/21270 (PMC8082389; doi:10.2196/21270)
Supplement: Multimedia Appendix 3 [file humanfactors_v8i2e21270_app3.docx]

Appendix 3. Usability Task Completion Exercises.

Please complete the following tasks using the InformedChoices Decision Aid, and respond either Yes or No when asked "Were you able to the complete this task?

1. Use the prognosis simulator to find an estimate of your CF progression over the next 1, 2, and 3 years. Yes or No

Were you able to complete this task?

1. Navigate to the page containing basic information about lung transplant. Yes or No

Were you able to complete this task?

1. Find resources for making an advance directive. Yes or No

Were you able to complete this task?

1. Find the values elicitation page for breathing tube. Yes or No

Were you able to complete this task?

1. Complete the exercise and see your results. Yes or No

Were you able to complete this task?

1. Find the page containing patient and caregiver stories about intubation and lung transplant. Yes or No

Were you able to complete this task?
