# Supplementary material for: Developing a Decision Aid to Facilitate Informed Decision Making About Invasive Mechanical Ventilation and Lung Transplantation Among Adults With Cystic Fibrosis: Usability Testing
Source: JMIR Hum Factors. 2021 Apr 14;8(2):e21270. doi: 10.2196/21270 (PMC8082389; doi:10.2196/21270)
Supplement: Multimedia Appendix 4 [file humanfactors_v8i2e21270_app4.docx]

Appendix 4. Usability Testing Survey.

Flow/Ease of Navigation

1. Overall, was it easy to go through the website to find the information you were looking for? Yes or No

2. Were the pages providing sliding scales to rate your preferences for getting a breathing tube/lung transplant easy to use? Yes or No

3. Were you able to use the prognosis simulator to find estimates for your CF progression over the 3 year time-period? Yes or No

4a. Did you have any difficulty getting the estimates? Yes or No

4b. How would you explain these estimates to a someone else?

4c. Do you think that these estimates are accurate? Why or why not?

5. Which visual representation of the estimates did you like the best for showing the likely course of your CF progression?

a. Graph (with the higher and lower range of values presented)

b. Percentages (basic numbers)

c. Icon arrays (little people)

6. Did you understand what the range of values found on the graph represented about your health?

a. I understood what it meant

b. I somewhat understood what it meant

c. I did not understand what it meant

7. Were you able to navigate to the page containing basic information about breathing tube? Yes or No

8. Were you able to find the information you needed about breathing tubes? Yes or No

9. Was the page easy to find and read through? Yes or No

10. Were you able to find the expanded information about breathing tubes? Yes or No

11. Did the section contain the right amount of information about breathing tubes?

a. Too Much

b. Too Little

c. Just Right

12. Were you able to find resources for making an advance directive? Yes or No

13a. Did you have any difficulty find these resources? Yes or No

13b. If yes, please explain.

14. Were you able to find the page providing sliding scales to rate your preferences for getting a breathing tube (What’s Important to Me page)? Yes or No

15. Were you able to view your results? Yes or No

16a. Did you have any difficulty getting the results? Yes or No

16b. If yes, please explain

17a. Do you think that the patient and family member stories about intubation and lung transplant are helpful for decision making helpful? Yes or No

17b. Why or why not?

18. Which did you prefer – listening to the audios or reading the stories?

a. Listening to the audios

b. Reading the stories

19. What other kinds of stories would you find helpful for making decisions about either intubation or lung transplant?

20. Did you prefer the shorter versions or the longer versions?

a. The shorter versions

b. The longer versions

Content/Readability

21. Did you find the information in the decision aid easy to understand? Yes or No

22. Were there any words/terms that you didn't understand? Yes or No

23. If yes, what words did you have difficulty understanding?

24. Was the tone of the information in the decision aid website appropriate? Yes or No

25. If no, why not?

Style/Design

26. Was the font easy to read? Yes or No

27. Were the colors...?

a. Too Bright

b. Too Dull

c. Just Right

28a. Did you like the graphics/visuals on the welcome page? Yes or No

28b. Why or why not?

29. What was your reaction to viewing the images of the intubated patient?

30. Was there enough space between paragraphs and questions throughout the decision aid (in other words, too much or too little white space)?

a. Too Much

b. Too Little

c. Just Right

31. Were there any directional cues that needed to be added? For example, did you know/understand how to navigate through the site? Yes or No

32. What were the top 3 things that you remember about the decision aid? (Please number your responses).

33. What areas of the decision aid did you find to be most helpful for decision making about and CF advance care planning (Check all that apply)?

a. Prognostic estimates calculator

b. Values preferences exercises

c. Patient and caregiver stories about lung transplant and breathing tubes

d. Risk/Benefits comparisons

e. Descriptions of each procedure

f. Links to further reading

g. Links to advance care planning tools

34. Please elaborate on your answer

35. Do you have any suggestions or recommendations for improving the decision aid? Please describe.
