# Supplementary material for: Developing a Decision Aid to Facilitate Informed Decision Making About Invasive Mechanical Ventilation and Lung Transplantation Among Adults With Cystic Fibrosis: Usability Testing
Source: JMIR Hum Factors. 2021 Apr 14;8(2):e21270. doi: 10.2196/21270 (PMC8082389; doi:10.2196/21270)
Supplement: Multimedia Appendix 5 [file humanfactors_v8i2e21270_app5.docx]

Appendix 5. Clinicians Demographics

1. What is your age?
2. What is your gender?
   1. Male
   2. Female
3. What is your profession?
   1. Physician
   2. Nurse Practitioner
   3. RRT
   4. Social Worker
4. How many years have you been seeing CF patients?
5. What is your race/ethnic background?
   1. White
   2. Black/African-American
   3. Hispanic or Latino
   4. Asian/Asian-American
   5. Other
6. What is your current religious affiliation?
7. Were you born in the US? Yes or No
