# Supplementary material for: Developing a Decision Aid to Facilitate Informed Decision Making About Invasive Mechanical Ventilation and Lung Transplantation Among Adults With Cystic Fibrosis: Usability Testing
Source: JMIR Hum Factors. 2021 Apr 14;8(2):e21270. doi: 10.2196/21270 (PMC8082389; doi:10.2196/21270)
Supplement: Multimedia Appendix 6 [file humanfactors_v8i2e21270_app6.docx]

Appendix 6. Patient and Surrogate Demographics

1a. How old are you?

Or

1b. How old is the CF person to whom you provide care?

2a. Do you have CF? Yes or No

Or

2b. If you do not have CF, are you the caregiver or someone with CF? Yes or No

3a. What is your current FEV1 level?

Or

3b. What is the current FEV1 level of the CF person you are caring for?

4a. Do you have an advance directive? Yes or No

Or

4b. Does the CF person you are caring for have an advance directive? Yes or No

5a. Have you ever been intubated? Yes or No

Or

5b. Has the CF person you are caring for ever been intubated? Yes or No

6a. Have you ever had a lung transplant? Yes or No

Or

6b. Has the CF person you are caring for had a lung transplant? Yes or No

7. What is your relationship/marital status?

a. Single

b. Married

c. Divorced/Separated

d. Widowed

e. Other

8. What is your gender?

a. Male

b. Female

9. What is your race/ethnic background?

a. White

b. Black/African American

c. Hispanic/Latino

d. Caribbean/West Indian

e. Other

10. What is your current religious affiliation?

a. Christian

b. Jewish

c. Muslim

d. Other

e. None

11. What is your current employment status?

a. Employed Full Time

b. Employed Part Time

c. Self-Employed

d. Retired

e. Unemployed

12. What is the highest level of education you have completed?

a. Less than 8^th^ Grade

b. Less than 12^th^ Grade

c. High School

d. Some College

e. College – BA, BS, etc

f. Post College – Masters, PhD, etc.

13. What would you say is your current economic class?

a. Lower Class

b. Lower Middle Class

c. Middle Class

d. Upper Middle Class

e. Upper Class

14. Were you born in the US? Yes or No

15. If you were not born in the US. How long have you been living in the US?

16. What language(s) do you speak at home?

a. English

b. Spanish

c. French/Creole

d. Chinese

e. Other

17. Do you use an interpreter when you see the doctor? Yes or No

18. If you use an interpreter, who is the interpreter?

19. If you do not use an interpreter, would you like to use an interpreter? Yes or No

20. Do you currently have access to a computer with internet in your home? Yes or No

21. How comfortable are you using the internet?

a. Not at all comfortable

b. Somewhat uncomfortable

c. Mostly comfortable

d. Extremely comfortable

22. On average, how many hours per week do you spend on the internet?

a. Never

b. Less than 5 hours per week

c. 5 to 10 hours per week

d. 10 to 30 hours per week

e. Over 30 hours per week

23. What type of insurance do you have?

a. Public insurance (Medicaid, Medicare, VA, etc.)

b. Private insurance (United, BlueCross, etc.)

c. No insurance

d. Not sure

24. What is your current living arrangement?

a. Live alone

b. Live with a spouse or partner

c. Live with another family member

d. Other

25. How would you rate your health in general?

a. Excellent

b. Very Good

c. Good

d. Fair

e. Poor

26. Considering all parts of your life (or the CF person you are caring for) – physical, emotional, social, spiritual, and financial – over the past two (2) days, how would you rate the quality of your life on a scale of 0-100 with 0 being very bad and 100 being excellent?

27. Considering all parts of your life (or the CF person you care for) – physical, emotional, social, spiritual, and financials – two (2) weeks prior to your hospital admission, how would you rate the quality of your life?

28. Has a clinician ever discussed planning in the event of a potentially life threatening situation resulting from CF? Yes or No
